# Seasonal influenza vaccination delivery through community pharmacists in England: evaluation of the London pilot

**DOI:** 10.1136/bmjopen-2015-009739

**Published:** 2016-02-16

**Authors:** Katherine Atkins, Albert Jan van Hoek, Conall Watson, Marc Baguelin, Lethiwe Choga, Anika Patel, Thara Raj, Mark Jit, Ulla Griffiths

**Affiliations:** 1 Department of Infectious Disease Epidemiology, Faculty of Epidemiology and Population Health, London School of Hygiene and Tropical Medicine, London, UK; 2 Respiratory Disease Department, Public Health England, London, UK; 3 NHS England, London, UK; 4 Imperial GP Specialty Training, Department of Primary Care and Public Health, Imperial College London, London, UK; 5 Screening and Immunisations, London Region, Public Health England, London, UK; 6 Department of Global Health, Faculty of Public Health and Policy, London School of Hygiene and Tropical Medicine, London, UK

**Keywords:** PUBLIC HEALTH

## Abstract

**Objective:**

To evaluate the effectiveness and cost of the pan-London pharmacy initiative, a programme that allows administration of seasonal influenza vaccination to eligible patients at pharmacies.

**Design:**

We analysed 2013–2015 data on vaccination uptake in pharmacies via the Sonar reporting system, and the total vaccination uptake via 2011–2015 ImmForm general practitioner (GP) reporting system data. We conducted an online survey of London pharmacists who participate in the programme to assess time use data, vaccine choice, investment costs and opinions about the programme. We conducted an online survey of London GPs to assess vaccine choice of vaccine and opinions about the pharmacy vaccine delivery programme.

**Setting:**

All London boroughs.

**Participants:**

London-based GPs, and pharmacies that currently offer seasonal flu vaccination.

**Interventions:**

Not applicable.

**Main outcome measures:**

Comparison of annual vaccine uptake in London across risk groups from years before pharmacy vaccination introduction to after pharmacy vaccination introduction. Completeness of vaccine uptake reporting data. Cost to the National Health Service (NHS) of flu vaccine delivery at pharmacies with that at GPs. Cost to pharmacists of flu delivery. Opinions of pharmacists and GPs regarding the flu vaccine pharmacy initiative.

**Results:**

No significant change in the uptake of seasonal vaccination in any of the risk groups as a result of the pharmacy initiative. While on average a pharmacy-administered flu vaccine dose costs the NHS up to £2.35 less than a dose administered at a GP, a comparison of the 2 recording systems suggests there is substantial loss of data.

**Conclusions:**

Flu vaccine delivery through pharmacies shows potential for improving convenience for vaccine recipients. However, there is no evidence that vaccination uptake increases and the use of 2 separate recording systems leads to time-consuming data entry and missing vaccine record data.

**Strengths and limitations of this study**

- London-based pharmacy initiative is the largest evidence base on which to evaluate the potential of the national pharmacy flu vaccine provision programme roll out.
- No previous analysis of London-based pilot prior to national programme roll out.
- First analysis to provide effectiveness and economic evaluation across London-based pilot to inform national programme implementation.
- Results may not be generalisable to other areas of the country or the national pharmacy delivery programme.

## Introduction

The UK seasonal influenza (flu) immunisation programme aims to protect those who are most at risk of serious illness or death from flu infection and to reduce transmission of the infection, which contributes to the protection of vulnerable patients in whom vaccine efficacy is low.^1^ Available between September and January each year, the National Health Service (NHS) currently offers flu vaccination free of charge to: (1) anyone aged 65 years or older; (2) pregnant women at any stage of pregnancy; (3) long-stay care home residents; (4) those who are in receipt of a carer's allowance or those who are the main carer for an elderly or disabled person whose welfare may be at risk if they fall ill; (5) patients with chronic disease: chronic respiratory disease, chronic heart disease, chronic kidney disease, chronic liver disease, chronic neurological disease, diabetes, immunosuppression, and asplenia or dysfunction of the spleen; and (6) all other children aged 2, 3 and 4.^2^ The first five groups are offered inactivated vaccine, whereas children with no clinical risks are offered live attenuated vaccine. Until the 2013/2014 flu season, general practitioners (GPs) or nurses vaccinated all eligible individuals in London except in three administration areas where there were small pilot studies for pharmacy delivery.

To increase access and improve patient healthcare choice and opportunity, particularly within underserved communities, NHS England (London Region; NHS LR), via consultation with North East London Local Pharmaceutical Committee and Pharmacy London, began the ‘pharmacy initiative’ in 2013/2014, which enabled pharmacists to provide the seasonal flu vaccine to eligible individuals. Through this initiative, the NHS reimbursed pharmacies when they vaccinated an individual aged 13 years or older with inactivated flu vaccine belonging to any of the first five eligibility groups. From 2014/2015, the initiative was expanded to allow pharmacies to offer inactivated flu vaccines to clinically at risk children from aged 2 and older.

Community pharmacies offer vaccinations to both children and adults in a number of countries, including the USA^3^ and Canada.^4^ ^5^ A London-wide patient survey previously conducted by the NHS recorded a high level of patient satisfaction with the pharmacy initiative, with patients reporting that convenience played a critical role in their decision to get vaccinated at the pharmacy (see online supplementary information A).

NHS England recently announced that pharmacies nationwide would be able to offer flu vaccines to eligible patients for 2015/2016 flu season.^6^ ^7^ Thus, the London-based initiative has been the largest of its kind in England and, as such, can inform decisions about the current national roll out. In particular, to broaden the current pharmacy initiative to the rest of the country, the London pilot programme must demonstrate effectiveness and cost-effectiveness relative to the previous status quo of GP-only flu vaccine administration.

In this study, we evaluate the impact of expanding flu vaccine provision into community pharmacies within five key areas: (1) the impact on vaccination uptake; (2) impact on the reporting system, data collection and administrative burden; (3) pharmacy and GP opinions on the pharmacy initiative; (4) the impact on individual accessibility to flu vaccine services; and (5) the cost of flu vaccine administration. These results can directly inform the proposed nationwide programme by predicting outcomes and raising likely issues.

## Methods

### Data: Sonar and ImmForm data

To evaluate the uptake in pharmacies across London, we used data from Sonar, a pharmacy-reporting system detailing seasonal influenza vaccine administration by all participating pharmacies, from 2013/2014 and 2014/2015. Sonar data from 2014/2015 did not include stratification by risk group. Individuals classified in Sonar 2013/2014 as ‘frontline healthcare staff’ (7% of the 68 220 patients reported as attending a pharmacy for the flu vaccine), ‘householders of immunocompromised individuals’ (<1% of pharmacy patients) or ‘living in long-stay accommodation facilities’ (<1% of pharmacy patients) were not provided in the ImmForm, the analogous database used for vaccine reporting by GPs, and thus were excluded from the analysis. For each vaccine recipient or set of recipients, Sonar data records report the primary care trust (PCT) of the pharmacy where the vaccine was administered, and the PCT of the GP where the recipient is registered.

We used data on vaccine delivery reported by GP practices via the ImmForm system from 2010/2011 to 2014/2015, provided by NHS LR. Pharmacies are required to report any vaccine administration to the GP where their patient is registered (this information is provided by the patient). The GP practice is then required to enter this information into the ImmForm system. For 2014/2015, ImmForm data tally the vaccine doses administered outside the GP practice. Prior to the 2013/2014 season, ImmForm data stratified GP practice by PCT. In 2013/2014, when administrative areas were reclassified from PCTs to clinical commissioning groups (CCGs), the ImmForm data reflected this change. For ease of comparison, we present the data across areas by PCTs where applicable. All named areas were mapped identically from PCT to CCG with the following exceptions: West London (Kensington and Chelsea and QPP) CCG was mapped to Kensington and Chelsea PCT; Central London (Westminster) CCG was mapped to Westminster PCT; Merton CCG and Sutton CCG were combined to map to Sutton and Merton PCT; Richmond CCG was mapped to Richmond and Twickenham PCT. ImmForm data were stratified by inclusion criteria: (1) 65+ years, (2) carers <65 years, (3) pregnant women with no clinical risk factors, and (4) clinical (<65 years with diabetes, kidney disease, heart disease, immunosuppression, respiratory disease, neurological disease, liver disease.

### Data: pharmacy survey

We analysed results of a survey conducted by NHS LR involving 1230 pharmacies, which participated in the pharmacy initiative, between 5 and 16 March 2015 across London (see online supplementary information B; 5% (58) response rate). Data were collected on (1) pharmacy staff time use for flu vaccine procurement, administration and paperwork; (2) flu vaccine brand choice; and (3) opinions about the pharmacy initiative. Data were cleaned to remove any reported duration figures that were over 3 SDs away from the mean or were clearly typographical errors because they were non-numeric or an order of magnitude greater than the other responses.

### Data: GP survey

We analysed results of a survey conducted by NHS LR to all 1406 GP practices between 5 and 16 March 2015 across London (see online supplementary information C; 24% (344) response rate). Data were collected on (1) opinions about the pharmacy programme, (2) logistics of vaccine delivery, (3) vaccine brand choice. Data were cleaned to remove any responses that were unclear.

### Evaluating vaccine coverage and vaccine administration reporting

As there were only a very small proportion of individuals receiving their flu vaccine at a pharmacy who might have not been registered at a GP (<1%), we used the total registered individuals from the GP ImmForm data as the denominator in our calculations where appropriate.

GP ImmForm data were stratified by ages 16 to under 65 years, whereas Sonar data were stratified by ages 13 to under 65. Therefore, the total numbers eligible to receive the vaccine at a pharmacy marginally inflated as they included teenagers between 13 and 15 years. However, as the number of people eligible to receive the vaccine in this age bracket is likely extremely small, this effect would also be very small.

To evaluate whether the extent to which vaccine uptake in pharmacies is associated with high overall vaccine uptake, we explored the linear association with 2014/2015 flu vaccine uptake within PCTs as the dependent variable, and the previous season's uptake across PCTs and proportion of vaccine doses administered at a pharmacy in 2014/2015 as predictor variables.

To determine the completeness of reporting of flu vaccine administration in pharmacies in the GP database, we compared the vaccine dose uptake reported by Sonar 2014/2015 data with the vaccine uptake in locations other than the GP practice, as reported in ImmForm 2014/2015 data.

### Calculating vaccine delivery costs

We calculated the costs of the flu vaccine delivery through pharmacies from both the NHS and the pharmacy perspective and the costs of vaccine delivery through GPs from the NHS perspective. Investment costs were annualised by dividing the total costs by the expected lifetime of the resources. We assumed that services for waste disposal and sharps removal from both pharmacies and GPs were managed and paid for directly by the NHS. Thus, no additional cost was taken into account from either the pharmacy or NHS perspective.

#### Pharmacy vaccine delivery

From the NHS perspective, costs comprise the initial investment costs of establishing the programme and the recurrent reimbursement cost to pharmacies, as reported by NHS LR. The life expectancy of Sonar software was assumed to be 5 years. Recurrent costs for 2014/2015 were assumed to be the same as in 2013/2014. Pharmacies are reimbursed a fixed price by the NHS for vaccine purchase (£7.08 per dose, including value-added tax (VAT)) and administration (£7.51 per dose), irrespective of their choice of vaccine they wish to offer.

We estimated personnel and material costs from the pharmacy perspective using data from our pharmacy survey: the category of personnel working on the flu vaccine programme, the time spent by personnel on various tasks, whether any medical supplies are being used, such as plasters and cotton wool, and whether they have incurred any capital investment costs to facilitate flu vaccine delivery. Gross salaries for pharmacy staff categories were assigned as follows: assistant—£6.31/h (minimum wage); technician or dispenser—£6.93/h;^8^ pre-registration pharmacist—£18 440/year;^9^ pharmacist—£45 946 (inflated to London salaries from £38 610 by 19%).^10^

The depreciation time was assumed to be 10 years for electrical items (ie, refrigerator). We assumed that the pharmacies paid the list price for their vaccine supply and calculated this average cost from the list price of the vaccines^11^ and the distribution of vaccine brand choice (see online supplementary information B).

#### GP vaccine delivery

GPs are reimbursed the list price of the vaccine they choose to purchase (discounted by a percentage, ie, determined by the total monthly purchase reimbursement request^12^), a fixed service payment for vaccine administration (£7.64 per dose), and a dispensing fee. The dispensing fee also depends on the monthly number of doses administered by a clinic and whether the GP clinic is classified as a dispensing practice. With the average number of monthly doses less than 400 per clinic, we averaged the dispensing practice (230.9p) and non-dispensing practice (240.6p) reimbursement fees to get a dispensing fee of £2.25.^12^ We calculated the cost of GP flu vaccine delivery per dose as the sum of the cost of the GP vaccine service fee, the average dispensing fee and the average cost of vaccine purchase reimbursement from the NHS. The average cost of vaccine purchase reimbursement was calculated from the list prices of the vaccines (British National Formulary (BNF), 2015) and the distribution of vaccine brand choice by GPs as evaluated in the online survey (see online supplementary information C).

### Implementation of analysis

All analyses were conducted using MATLAB (R2014b. The MathWorks Inc., Natick, MA; 2014). Code used for analysis and de-identified data are available at https://github.com/katiito/PharmacyVaccination

The survey was administered by NHS LR on behalf of London School of Hygiene and Tropical Medicine (LSHTM), and the survey was coded using, and hosted by, Survey Monkey.

## Results

### The impact on vaccination uptake

**Figure 1:**
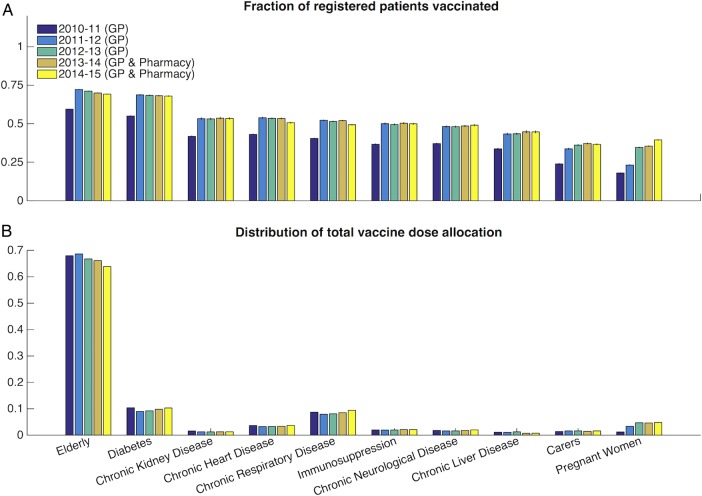
General Practitioner (GP)-reported vaccine uptake by risk group. (A) Vaccine dose allocation across both GPs and pharmacies as reported in ImmForm reporting system between 2010 and 2014. (B) The fraction of total doses administered per risk group.

There was no change in reported vaccine coverage across all risk groups between seasons 2011/2012 (60.1%) and 2012/2013 (60.4%) (t test, p=0.36), or between 2012/2013 (60.4%) and 2013/2014 (60.5%), the first year of the pan-London pharmacy initiative (t test, p=0.84; figure 1A). There was a slight reduction in reported vaccine uptake of 1.8–3.0% between 2013/2014 (60.5%) and 2014/2015 (58.1%; t test, p<0.001), and of 1.3–3.3% between 2012/2013 and 2014/2015 (t test, p=0.009; figure 1A), with the majority of doses administered to the elderly (figure 1B).

**Figure 2:**
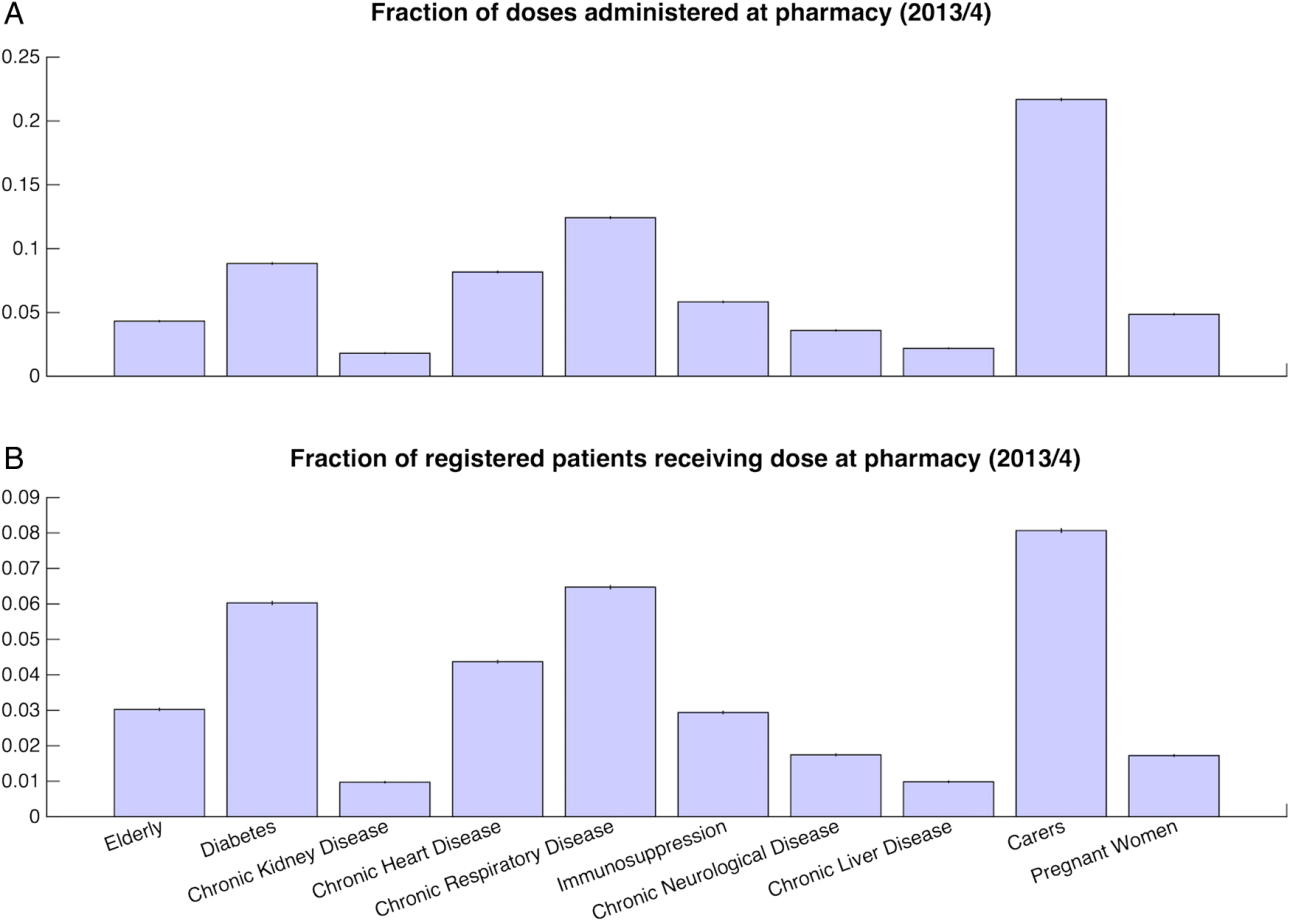
Pharmacy-reported vaccine uptake by risk group. (A) Probability that vaccine dose was administered at a pharmacy by risk group. (B) Total vaccine uptake in population at pharmacies across risk groups.

For 2013/2004, the vaccine uptake ranges between 35% for pregnant women and 70% for those aged 65 years and over (figure 1). The risk groups that increased their uptake between the 2012/2003 and 2013/2004 seasons (kidney disease, immunosuppression, respiratory disease, neurological disease, liver disease, carers and pregnant women) did so only by 1% or less. The probability that individuals received their vaccine in pharmacies varied between 2% (chronic kidney or liver disease) and 22% for carers (figure 2A). The probability that any individual within each group became vaccinated at a pharmacy stands between 1% for patients with kidney or liver disease and 8% for carers (figure 2B).

The fraction of vaccine doses administered at pharmacies ranged from 2% to 20% (see online supplementary figure S1A). Averaging across all administrative areas, there was a rise in the fraction of eligible people vaccinated at pharmacies, from 3.5% (3.1% to 3.9%) in 2013/2014 to 5.2% (4.8% to 5.6%) in 2014/2015 (see online supplementary figures S1B,C, S2). There was a significant increase in the proportion of flu vaccine doses administered at pharmacies between the first and second year of pan-London pharmacy initiative (t test, p<0.001, see online supplementary figure S1A,B). This increase is explained by both an increase in the number of participating pharmacies (from 718 in 2013/2014 to 1089 in 2014/2015), as well as a slight increase in the average number of doses administered at each pharmacy (95 in 2013/2014, with a total of 68 220 doses and 99 in 2014/2015, with a total of 108 186 doses).

### Impact on the reporting system, data collection and administrative burden

**Figure 3:**
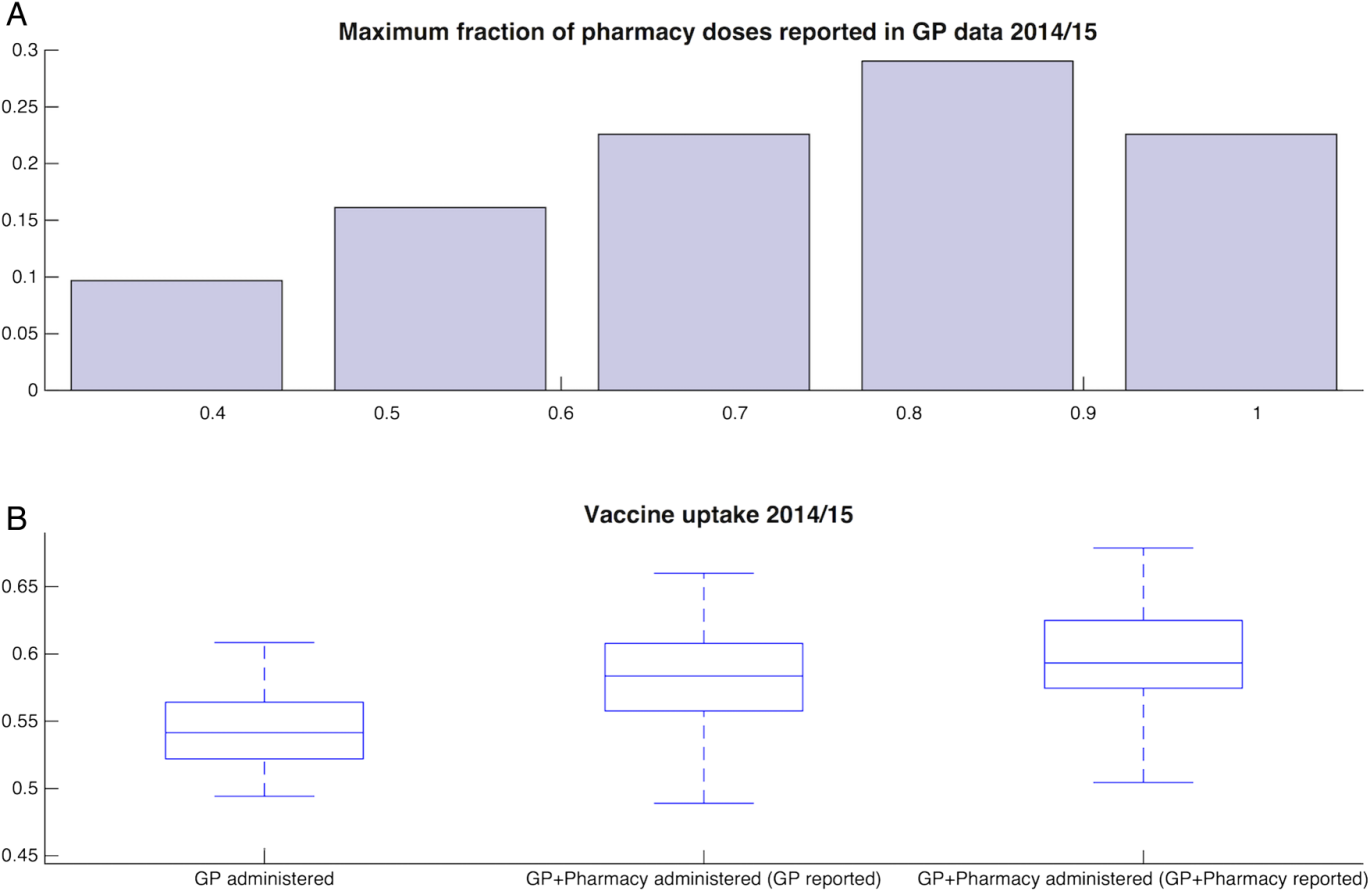
Completeness of reporting across all administrative areas. (A) Maximum estimate of fraction of pharmacy vaccine doses are subsequently recorded onto general practitioner (GP) recording system. (B) Partitioning 2014/2015 GP data by those vaccines administered in GPs, those administered in total as reported by GP recording system and those administered everywhere accounting for under-reporting in GP recording system.

We compared the 2014/2015 Sonar data that record the number of pharmacy-administered vaccines with 2014/2015 ImmForm data that record the number of vaccine doses delivered to GP-registered patients by healthcare professionals outside the GP practice. This comparison found that, at most, 75% of pharmacy-administered vaccine doses are subsequently reported in the ImmForm data across all PCTs (SD=20%, figure 3A). This fraction is an upper bound on the completeness of reporting because some of the vaccine doses that are recorded to be administered by a professional outside the GP practice are not necessarily administered by pharmacy staff.

Accounting for potential under-reporting in vaccine coverage and comparing the annual vaccine coverage before and after the introduction of the pharmacy initiative, we found that there was no change in vaccine coverage between years 2011/2012 (60.1%) and 2014/2015 (59.7%; t test, p=0.34) and a marginal decrease between years 2012/2013 (60.4%) and 2014/2015 (59.7%; t test, p=0.05; figure 3B).

### Pharmacy and GP opinions on the pharmacy initiative

Pharmacists reported much greater support for the pharmacy initiative than did GPs (see online supplementary figures S3A–C, S4A–F). Responses from the online survey showed that the vast majority of pharmacists thought that the pharmacy initiative eased the burden on the healthcare system (see online supplementary figure S3A) and only a minority of pharmacists were concerned that their lack of access to patient record data was inefficient or led to difficulties with healthcare provision (see online supplementary figure S3B). By contrast, there was an equal split between GPs as to whether the initiative increased or decreased the administrative burden on them (29–30%, respectively). However, 61% of GPs were concerned with a loss of patient data due to incompleteness of reporting (see online supplementary figure S3C). Further comments by GPs emphasise the issue with incomplete data provision by pharmacists and the requirement for manual data entry (see online supplementary information D). The vast majority of pharmacists believed that their service improves choice for patients (98%) and is more convenient for patients (97%). In contrast, GPs are less uniformly positive about the advantages of pharmacy vaccination, with 60% believing it improves choice for patients and 40% believing it is more convenient for patients. GPs were concerned with a reduced quality of healthcare provision for their patients (40–50%), issues with safety (40%) and personal financial loss to themselves (52%; see online supplementary figure S3). Most pharmacists were interested in increasing their vaccine provision capacity (91%, see online supplementary figure S4D). More information from the surveys is found in online supplementary figures S3, S4 and supplementary information D).

### The impact on individual accessibility to flu vaccine services

At least 99% of patients receiving the flu vaccine at a pharmacy were registered with a GP (table 1). For individuals who received their vaccine at a pharmacy, 24% (2013/2014) and 20% (2014/2015) did so in a PCT area where they were not registered with their GP (table 1).

**Table 1:** Total number of reported vaccines administered in pharmacies from Sonar

	2013/2014*	2014/2015*
Pharmacy is in the same PCT as the patient's GP	50 988 (75%)	86 282 (80%)
Pharmacy is in a different PCT as the patient's GP	16 640 (24%)	20 989 (19%)
Patient's GP is outside London	3221	3521
Patient's GP is not reported (null/unknown/none)	592 (1%)	915 (1%)
Total vaccine doses reported	68 220 (100%)	108 186 (100%)

*Per cent of total vaccine doses administered in pharmacy in parentheses.

GP, general practitioner; PCT, primary care trust.

### The cost of flu vaccine administration

#### NHS perspective

We calculated the annual cost per dose to the NHS (from the commissioner perspective) of pharmacy flu vaccination delivery to be £14.88 in the 2013/2014 season and £14.78 in the 2014/2015 season (table 2). The cost per dose decreases a little with the number of doses delivered due to greater utilisation of the fixed investment costs. Changing the life expectancy of the Sonar system from 10 to 2 years, the cost per dose in 2014/2015 varies between £14.75 and £14.89, respectively.

**Table 2:** NHS perspective costs

	Pharmacy delivery				GP delivery			
	Cost per dose		Total annual cost		Cost per dose		Total annual cost*	
	2013/2014	2014/2015	2013/2014	2014/2015	2013/2014	2014/2015	2013/2014	2014/2015
Recurrent costs								
NHS vaccine service payment	£7.51	£7.51	£512 332	£812 477	£7.64	£7.64	£7 638 296	£7 850 169
Vaccine purchase payment†	£7.08	£7.08	£482 998	£765 957	£7.24‡	£7.24§	£7 698 283	£7 911 819
Dispensing fee	NA	NA	NA	NA	£2.25	£2.25	£2 249 498	£2 311 895
Sonar service fee	£0.18	£0.12	£12 500	£12 500¶	NA	NA	NA	NA
Investment costs								
Sonar development	£0.11	£0.07	£7200§	£7200¶	NA	NA	NA	NA
Total	£14.88	£14.78	£1 015 030	£1 598 134	£17.13	£17.13	£17 456 316	£17 897 611

*Includes all patients 65 and over, carers under 65 years, at risk individuals 16–65 years.

†Includes 20% VAT for each dose reimbursed.

‡Calculated as average vaccine list price with VAT (£7.70) less a discount of 5.93% based on 340 doses per clinic per month.^12^

§Total cost (£36 000) is depreciated over 5 years.

¶Total cost figures for 2013/2014 used.

GP, general practitioner; NA, not available; NHS, National Health Service.

The annual cost to the NHS of GP flu delivery was found to be £17.13 per dose (table 2). To calculate the reimbursed price per dose to the GP, we discounted the vaccine purchase price by 5.93%, based on a list vaccine price of £7.70 with on average 340 monthly doses purchased per clinic. If more patients were diverted from GPs to become vaccinated in pharmacies, the costs of GP delivery per dose would increase and the costs of pharmacy delivery per dose would decrease, relative to the respective investment costs.

#### Pharmacy perspective

The vast majority of pharmacists reported that they spend less than half an hour purchasing vaccine stock for the season, and half of the pharmacists spent less than 1 h on reimbursement paperwork over the season (see online supplementary figure S5A,B). Most of the vaccine-related pharmacist time is spent with patients, with a 17 min mean consultation time (see online supplementary figure S5C). The mean time spent inputting data and sending patient information to GPs was reported as 7 min (see online supplementary figure S5D).

**Figure 4:**
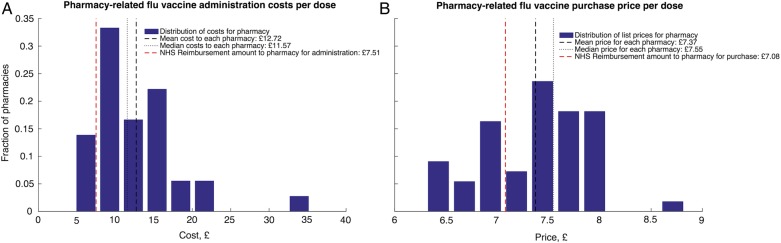
Pharmacy delivery costs. Total administration costs and vaccine purchases prices for pharmacy flu vaccine delivery in 2014/2015 from pharmacy and National Health Service (NHS) perspectives. Pharmacy survey data are only included in calculation when all cost and time use data are present in order to calculate the total cost for each pharmacy.

The cost of the time spent with the patient receiving the vaccine accounts for 65% of the total cost to the pharmacist (table 3). The cost of time spent on data input and GP paperwork accounts for a further 21% of the total cost to the pharmacist. The average salary cost of vaccine delivery for a pharmacist is £12.72 per dose for 2014/2015, with 50% of pharmacists spending £11.57 per dose or less (table 3 and figure 4A). The average list price for the vaccines chosen by the pharmacists is £7.37 (figure 4B, see online supplementary figures S5b, S6). Combined, the average pharmacist cost to deliver a flu vaccine is £20.09 per dose, £5.51 less than they are reimbursed. Less than 1% of the pharmacists reported offering the most expensive vaccine, the tetravalent preparation (Fluarix Tetra).

**Table 3:** Pharmacy perspective average costs for vaccine administration and purchase

	Number of pharmacies incurring cost	Cost per year per pharmacy if cost incurred	Average pharmacy cost per season		Average pharmacy cost per dose‡	
			2013/2014	2014/2015	2013/2014	2014/2015
Recurrent costs						
Plasters	34/58	£1.75	£1.03	£1.03	£0.01	£0.01
Procurement time (excluding brand choice)	All	NA	£8.18	£8.18	£0.09	£0.08
Reimbursement paperwork time	All	NA	£121.87	£121.87	£1.28	£1.23
Patient time	All	NA	NA	NA	£8.11	£8.11
Data input time	All	NA	NA	NA	£2.66	£2.66
Investment costs						
Refrigeration	6/58	£57.00	£5.90	£5.90	£0.06	£0.06
Admin total (with data above)					£12.79	£12.72
Admin total (with 1 min data input time)					£10.61	£10.54
Vaccine purchase list price					£7.37	£7.37
Total (with reported data)					£20.16	£20.09

*Calculated using a cost of £30,000+ 20% VAT (personal communication NHS LR).

†Base case costs include pharmacist salaries inflated by 19% for London costs.^10^

‡When per season cost is given, per dose cost calculated by dividing per season cost by 95 (2013/2014) or 99 (2014/2015; average number of doses delivered by each pharmacists in each season).

NA, not available; VAT, value-added tax.

## Discussion

This study evaluates the costs and benefits of the pan-London seasonal influenza programme offering eligible individuals their flu vaccine in community pharmacies as an alternative to local GP delivery. To assess flu vaccine coverage across London before and after the start of the pharmacy initiative, we analysed two data sets, ImmForm (GP reports) and Sonar (pharmacy reports). To evaluate the London-specific costs from the NHS and pharmacy perspectives of seasonal flu vaccination in pharmacies and GPs and the opinions of vaccine providers, we conducted an online survey administered to pharmacists and GPs across London. Our results do not show any evidence for increased vaccine uptake as a result of the pan-London flu vaccine pharmacy initiative. The cost to the NHS per vaccine dose of pharmacy delivery for the London initiative was lower than that of GP delivery. While we found evidence that the programme offered convenience and choice to patients, we found several disadvantages of the programme, most notably the incomplete reporting of pharmacy vaccine delivery into both recording systems.

To calculate incomplete reporting of vaccine delivery in the GP ImmForm system, we compared the number of vaccine doses that were noted to be delivered at somewhere other than the GP practice with the number of doses delivered to patients at pharmacies. If the place of vaccine dose administration was not correctly registered in ImmForm, then our results will have underestimated the number of vaccine doses that are recorded in ImmForm. Nevertheless, this potential issue further highlights the inefficient and information loss-prone nature of maintaining two recording systems with manual entry. Moreover, it is unclear how pharmacies ascertain whether individuals are eligible for vaccination. For a national programme with increased pharmacy vaccine uptake to remain efficient, it will be important to easily identify patient eligibility. For a national system, it may be helpful to identify advantages and failings of other vaccination reporting software, such as the Information Immunization System (IIS) used in the USA.^13^

Previous studies suggest that pharmacy-provided vaccines may increase uptake of seasonal influenza vaccine in high-risk groups. In a survey among 503 high-risk, non-elderly people in a pilot in Sheffield, 19% of vaccine recipients at a pharmacy stated that they would not have received the vaccine otherwise,^14^ an estimate consistent with a similar Canadian study.^5^ In our study, if 19% of people who received the vaccine at a pharmacy, that is, 19% of 10% of the total eligible population, would not have done so at their GP, the overall increase in vaccine coverage would be less than 2%. Such a change would likely be too small a difference to detect against the background fluctuations in coverage. We calculated the vaccine coverage for all seasons from ImmForm data. The true vaccine coverage is likely to be higher due to evidence of under-reporting of pharmacy-administered doses in ImmForm data. However, even accounting for these changes, our results remain consistent. Nonetheless, our analysis does not include the possibility of a secular trend of decreasing vaccine uptake in GP practices, which may obscure increases in coverage because of pharmacy administration. Moreover, it is likely that over time, pharmacy-administered vaccines will become more widely known and a rise in coverage observed. Our surveys indicate that pharmacy patients report convenience as the main factor in the decision to seek the flu vaccine at a pharmacy rather than their local GP, a finding consistent with other studies. For instance, people trust pharmacists to administer vaccines,^4^ ^15^ they are happy with the vaccination service they receive^5^ ^14^ and they believe that pharmacies offer a convenient way to receive the vaccine.^14^ ^16^ Indeed, one study reports that some people would rather pay out of pocket at the pharmacy instead of receiving the vaccine for free in primary care, largely due to convenience and accessibility.^16^ In our analysis, we observed a relatively high use of the pharmacy among carers. This result was also seen in a pilot programme using community pharmacies on the Isle of Wight, where there was an increase in uptake among carers and healthcare workers.^17^ A higher uptake among these groups (under 65 years and healthy) was perhaps expected as in the USA it has been shown that people who use the out-of-hours service provided by the community pharmacists (evenings and weekends) mostly consist of those under 65 years, and those without clinical conditions.^3^ This result suggests that the group using pharmacies the most are healthy individuals for whom vaccination allows only indirect protection of the elderly or sick. To streamline this route of vaccination, it may be possible to provide pharmacy vouchers with carer passports. Conversely, low pharmacy uptake for risk groups with historically low vaccine uptake, such as individuals with liver disease, neurological disorders or immunosuppression, provides increasing inequity for these groups.

Studies that have previously assessed physician opinion of regarding non-physician administered vaccine provision in the USA are broadly consistent with our findings: with between 60% and 70% of physicians agreeing that pharmacists and other providers have adequate training and provide a more convenient service to the patients.^18^ Moreover, 70% of physicians report incomplete vaccine documentation as a result of non-physician vaccine provision,^18^ and 90% of physicians have reported that this incomplete documentation is a barrier to patient referral.^19^

Promotion of the pharmacy initiative to eligible patients was agreed between NHS England and a steering group from London Pharmaceutical Committee (LPC). Promotion was predominantly by posters displayed in pharmacy windows, most of which were designed by the LPC, but some pharmacies used general pharmaceutical company vaccine campaign posters. Complementary outreach was achieved by other methods including website promotion (http://www.myhealthlondon.nhs.uk), twitter, photo shoots (eg, deputy Mayor of London receiving flu vaccine) and promotion via NHS trust occupational health departments.

Our results suggest that if the reported time use data in the surveys are accurate and the purchase price of vaccines is at list price, then London pharmacists would have needed to recoup £5.51 in order to break even on flu vaccine provision. There are three reasons how this shortfall is either recouped or non-existent. First, the actual time spent administering the vaccine or completing the data input may be less than reported. For example, under the assumption that only 1 min per dose is spent inputting data, the amount needing to be recouped by pharmacists drops by £2.18–£3.33 (table 3). As pharmacies adapt to the administration system, the duration of their patient consultation will likely decrease. For example, reducing the total time for vaccine administration and data input by 40% would allow pharmacies to break even. Moreover, the time required by pharmacists to administer and complete data entry per dose seems high relative to flu vaccinations in clinics,^20^ suggesting that bottom-up costing is likely to overestimate the costs of vaccine delivery. Overestimation of time spent administering vaccination or recording administration may arise due to the perception among pharmacists that our survey may influence remuneration decisions. Second, in practice, pharmacists will likely not pay the list price of their chosen vaccine, instead negotiating directly with the manufacturers for the best deal. For instance, if pharmacists wanted to minimise their expenditure based solely on the list price, they would choose Influvac (Abbott) retailing at £5.22 per dose, leaving a profit after reimbursement of £0.68. Thus, as many other brands were chosen, the negotiated price for pharmacy stock must provide more than a £0.68 per dose advantage. Third, pharmacists will hope to generate income by the increased footfall in their pharmacy. Even if the total cost is not recouped, the pharmacy may be willing to pay for the benefit of developing stronger patient confidence in their healthcare provision. We did not attribute any additional recurrent or investment costs of the GP delivery programme above the flu vaccine service fee and the purchase reimbursement price. Hence, the costs of the GP programme is likely underestimated as we did not include the costs of any flu communication material provided to GPs and the costs of managing vaccine data supplied by GPs. However, the cost of the pharmacy option may also be underestimated as it does not include additional costs for GP practices to input data from pharmacists, or contact patients who have already received the vaccine but are not recorded on the system. This latter cost may be relatively high due to the level of under-reporting of pharmacy uptake on the GP ImmForm system. The majority of vaccine doses in GP practices are administered by nurses or healthcare assistants, and as the salary of a nurse in a GP practice is only two-thirds of a pharmacist^10^ and even less for a healthcare assistant, and most of the vaccine delivery cost results from healthcare staff time, one would expect personnel costs for GP-based vaccine administration to be less costly than those for pharmacy-based vaccine administration.

Many GPs currently offer a call reminder service for the 65 years and over and a call and recall reminder service for other risk groups. If the costs for this reminder service are recouped in the service fee reimbursed to GPs per patient vaccinated, then decreasing the number of reimbursed service fees but maintaining the full call service for all eligible patients increases GP practice costs per vaccine dose. As GPs are paid for the vaccination coverage they achieve (irrespective of whether the practice delivered the vaccines themselves) under the Quality and Outcomes Framework (QOF), GP practices have an incentive to report complete uptake figures via ImmForm. However, there is no similar incentive to ensure complete reporting of pharmacy-delivered vaccine doses to GP practices. Moving to a single recording database system that is accessible to both pharmacists and GPs would align the incentives of recording by pharmacies for reimbursement with recording for complete patient health records. In the London pharmacy initiative, GPs and pharmacists received vaccine purchase reimbursement from the NHS differently; while GPs receive the list price for whatever vaccine stock they use, pharmacists receive a fixed fee regardless of the brand chosen. This reimbursement structure provides different incentives for vaccine brand choice. Leaving aside the health-related preferences of the healthcare provider, GPs would financially benefit from achieving a difference between the list vaccine price and the negotiated vaccine price. Pharmacists would also benefit from a large difference between the list vaccine price and the negotiated vaccine price, but any profit they receive would ultimately be contingent on the negotiated price to reimbursement fee difference. Therefore, with a fixed fee reimbursement contract, pharmacists will have less incentive to offer the more expensive quadrivalent vaccine (Fluarix Tetra). Indeed, across London, <1% of pharmacists offered the Fluarix Tetra compared with 5.5% of GPs. Previous proposals by the Department of Health have sought to procure flu vaccines centrally to streamline the vaccine programme and reduce vaccine purchase costs have been met with resistance by GPs.^21^ The current national pharmacy programme is equally unpopular with GPs,^6^ who are concerned with loss of income. Commissioning any re-evaluated vaccine programme is subject to various trade-offs: most notably maintaining incentives for vaccine provision, keeping vaccine purchase and reimbursement costs low, and ensuring high quality and complete reporting. Further, it is possible that there will be emerging issues when scaling up the pharmacy programme to a national level: more premises offering flu vaccines—such that the number of doses offered per premises decreases, coupled with the potentially opportunistic nature of pharmacy vaccination will likely result in more wasted flu doses per box compared with a limited number of providers, with a stable number of patients.

The ‘Community Pharmacy Seasonal Influenza Vaccination Advanced Service’, allowing pharmacies to administer flu vaccines, has been introduced in autumn/winter 2015.^22^ The programme differs from the pan-London initiative in a number of key ways: first, pharmacists are reimbursed for vaccine purchase in the same way as GPs, such that they claim for the list price (plus VAT) of the vaccine they choose to administer; second, they receive the same service payment as GPs of £7.64 per dose; third, pharmacists are paid an additional £1.50 per dose to cover training, revalidation and waste disposal.^23^

This study is subject to several limitations that must be considered before generalising the results countrywide. First, the coverage and survey data were London-specific, and these may not be representative of the country. Second, the survey response rate was very low, particularly for pharmacists. Such a low response may be indicative of a biased sample, and more importantly, not provide a large enough sample size for precise estimates of costs, which are calculated from self-reported activity durations. To achieve more accurate estimates of costs from all perspectives, it may be beneficial to conduct a larger, nationwide time use survey. Third, survey responses and coverage data are from 1 or 2 years, respectively. While more data may provide a more precise estimate of outcomes, it is also worth noting that a longer period of pharmacy-based vaccine delivery may increase the awareness of such a programme, and therefore ultimately vaccine coverage may increase over time. Furthermore, the analysis did not account for any underlying secular trends in the uptake of flu vaccination that may obscure any increase due to the pharmacy initiative. Fourth, the survey did not question patients and, notwithstanding the previous patient survey in London, there may be some interesting aspects of pharmacy delivery that have not been reported. For example, our GP survey found that GPs were concerned about the loss of healthcare opportunity for their patients, but given that nurses are more likely to administer flu vaccines in GP surgeries, and that pharmacists were probably more likely to have more time to administer the jabs, this may be a concern not echoed for patients. Finally, surveys were carried out retrospectively, and thus pharmacists may be prone to misreport activity durations on time use surveys. A more accurate survey would be conducted through the vaccination season in real time.

In summary, our findings indicate that the total cost per dose of seasonal flu vaccination is slightly lower when it is administered by pharmacies compared with GPs. However, we found no evidence of increased vaccine coverage as a result of the pharmacy initiative. Further, our results suggest that maintaining two reporting systems for GPs and pharmacies leads to substantial under-reporting of vaccine uptake and unreliable GP vaccine uptake data.
